# Relationships between motor patterns and intraluminal pressure in the 3-taeniated proximal colon of the rabbit

**DOI:** 10.1038/srep42293

**Published:** 2017-02-14

**Authors:** Xiaojing Quan, Zixian Yang, Mai Xue, Ji-Hong Chen, Jan D. Huizinga

**Affiliations:** 1 Department of Gastroenterology and Hepatology, Renmin Hospital of Wuhan University, Key Laboratory of Hubei Province for Digestive System Diseases, Wuhan, Hubei Province, China; 2 Farncombe Family Digestive Health Research Institute, McMaster University, Department of Medicine, Hamilton, ON, Canada

## Abstract

Manometry is used worldwide to assess motor function of the gastrointestinal tract, and the measured intraluminal pressure patterns are usually equated with contraction patterns. In the colon, simultaneous pressure increases throughout the entire colon are most often called simultaneous contractions, although this inference has never been verified. To evaluate the relationship between pressure and contraction in the colon we performed high-resolution manometry and measured diameter changes reflecting circular muscle contractions in the rabbit colon. We show that within a certain range of contraction amplitudes and frequencies, the intraluminal pressure pattern faithfully resembles the contraction pattern. However, when the frequency is very high (as in fast propagating contractions in a cluster) the consequent intraluminal pressures merge. When the contraction speed of propagation is very fast (above ~5 cm/s), the resulting pressure occurs simultaneous throughout the colon; hence simultaneous pressure is measured as are caused by fast propagating contractions. The very slow propagating, low amplitude haustral boundary contractions show a very characteristic pattern in spatiotemporal contraction maps that is not faithfully reproduced in the pressure maps. Correct interpretation of pressure events in high-resolution manometry is essential to make it a reliable tool for diagnosis and management of patients with colon motor dysfunction.

Manometry is widely used to assess motor function of the gastrointestinal tract; it measures intraluminal pressure activity using pressure sensors. Pressure sensing can be done using water-perfused catheters, solid-state sensors or a fiber optics catheter. It is almost always assumed that the pressure pattern is equivalent to a motility pattern. This is reflected in the fact that the pressure patterns are often called contraction patterns such as in High Amplitude Propagating Contractions; Retrograde Propagating Contractions and Simultaneous Contractions^1^. Logically, the “simultaneous contractions” were thought to be involved in slowing transit and facilitating mixing^2^, although we recently showed that that simultaneous pressure waves in the human colon are associated with gas expulsion and internal anal sphincter relaxation^3^. The question we asked was: are simultaneous pressure waves really associated with simultaneous contractions? Does the whole colon go into a spastic contraction all at once? This is of critical importance since motor patterns are evaluated to assess colon function or dysfunction^3^,^4^,^5^. Therefore our objective became to investigate the relationships between different colon motor patterns and intraluminal pressure. We measured diameter changes reflecting circular muscle contractions and we performed high-resolution manometry to assess pressure changes in the proximal rabbit colon that has taeniae and haustra in common with the human colon.

The rabbit colon has two motor patterns that are associated with propulsive activity, long distance contractions (LDCs) and fast propagating contractions. LDCs were first described as such in the rat colon^6^,^7^,^8^. They are one of the motor patterns collectively called Colonic Migrating Motor Complexes. The LDCs have a characteristic triangular shape in spatiotemporal maps and propagate at 0.8–2 cm/s in the rabbit colon^9^. The rabbit colon also exhibits fast-propagating contractions that are circular muscle ring contractions occurring at a high frequency (10–25 cycles/min) with a propagation velocity of ~6 cm/s, usually in antegrade direction, often appearing near simultaneous along the colon^10^. They may occur in clusters and are then associated with propulsion of content. Our hypothesis was that these two very distinct motor patterns, might reveal relationships between contraction and pressure development. Preliminary data have been reported in abstract form^11^,^12^.

## Results

### Pressure patterns as a consequence of Long Distance Contractions (LDCs)

**Figure 1:**
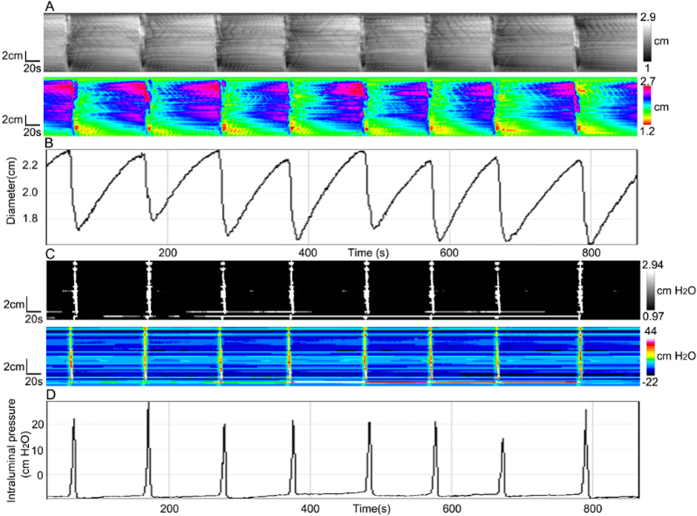
Long distance contractions (LDCs). (**A**) Spatiotemporal diameter map created from video recording of LDCs of the rabbit 3-taeniated intact colon. In these and subsequent figures two maps are shown, one in gray scales and one in a spectrum of colours. (**B**) Amplitude profile (diameter change) along the whole colon over time, from **A**. (**C**) Typical pressure patterns created from high-resolution manometry, as a consequence of the LDCs in **A**. (**D**) Intraluminal pressure profile along the whole colon over time, from **C**.

**Figure 2:**
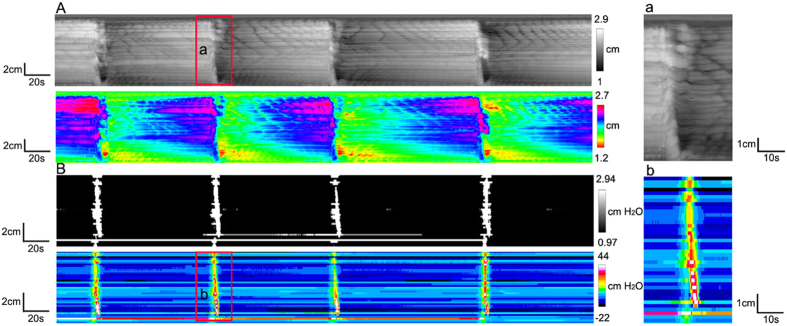
Propagation of LDCs. (**A**) Spatiotemporal diameter map of LDCs showing propagation at a speed of 2.1 ± 0.3 cm/s from proximal to distal. The red boxed area (a) shows an obvious peristaltic wave, enlarged at right. (**B**) Clear propagations were shown in pressure maps of LDCs. A zoomed in portion (b) on the right side of the pressure map shows an obvious antegrade propagation with an average velocity of 1.8 ± 0.4 cm/s.

LDCs were the most forceful propulsive motor activity at a frequency of 0.63 ± 0.17 cycles/min in the rabbit colon with a reduction in colon diameter of 0.56 ± 0.04 cm (N = 4). This was accompanied by intraluminal pressure of 37.4 ± 6.5 cmH_2_O (Fig. 1B and D). Motor activity and pressure activity were propagating at a velocity of 1.9 ± 0.2 cm/s and 1.7 ± 0.6 cm/s respectively (Fig. 2A and B) which is not statistically different.

The rate of rise of a developing LDC was much higher compared to the rate with which the contraction faded; in contrast, the rate of rise and rate of decline of the accompanying pressure transient were similar. As a consequence, the average duration of LDCs ranged from 20 s to 137 s (mean 71 ± 43 s), while the duration of pressure transients was 11 ± 2 s (N = 4, n = 24).

### Pressure patterns as a consequence of ripples

Ripples are a contraction pattern that is governed by interstitial cells of Cajal associated with the submuscular plexus (ICC-SMP)^10^. The ripple frequency ranged from 4.8 to 8.5 cycles/min (mean 6.6 ± 1.4 cycles/min). The propagation was too fast to be accurately measured. The reduction in diameter was 0.18 ± 0.02 cm (N = 8).

**Figure 3:**
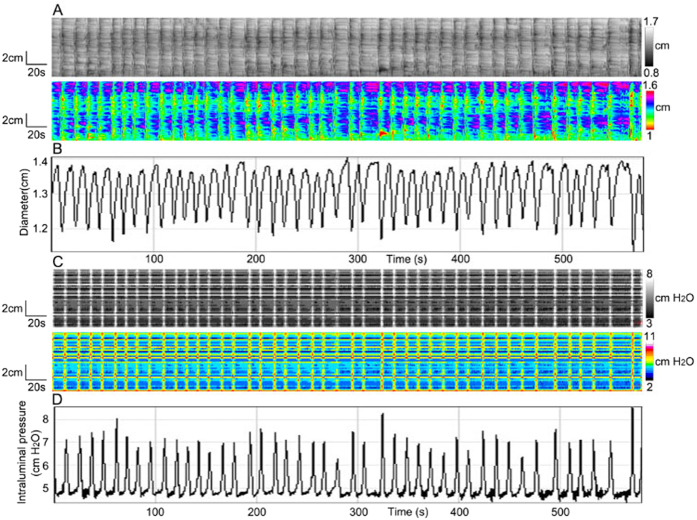
Ripples. (**A**) Spatiotemporal diameter maps of ripples of the whole rabbit proximal colon. (**B**) Amplitude profile along the whole colon over time, from **A**. (**C**) Pressure patterns associated with the ripples shown in **A**. (**D**) Intraluminal pressure profile along the colon over time, from **C**.

The pressure map shows pressure activity at the same frequency as the contractions (Fig. 3C). In some experiments ripples were interacting with haustral boundary contractions, and encounters between the two did not cause annihilation, the frequency of ripples was still constant, but the values of diameter and intraluminal pressure often had a strong waxing and waning appearance, which will be discussed later. The intraluminal pressure value was 2.5 ± 0.4 cmH_2_O. Hence, relatively low amplitude, fast propagating ripples caused clearly identifiable simultaneous pressure waves.

### Pressure patterns as a consequence of fast propagating contractions

**Figure 4:**
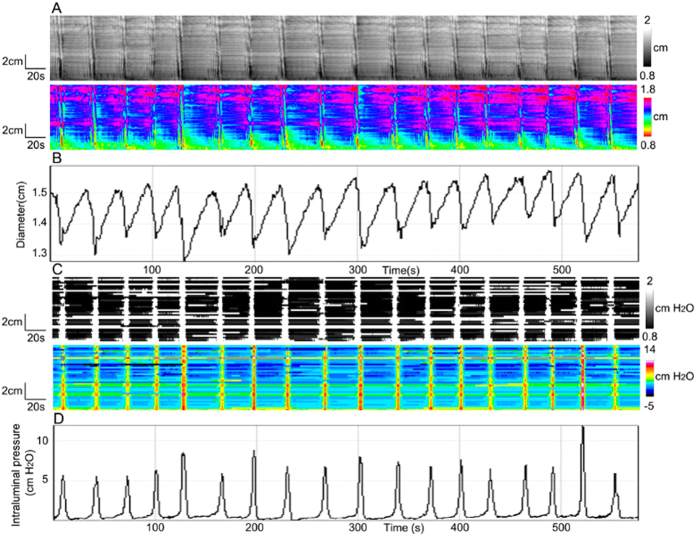
Fast propagating contractions. (**A**) Spatiotemporal diameter maps showing spontaneous fast propagating contractions at an average frequency of 20 ± 4 cycles/min, appearing in clusters, at a cluster frequency of 1.7 ± 0.5 cycles/min. (**B**) An amplitude over time profile of the clusters of fast propagating contractions in **A**. (**C**) Pressure patterns resulting from the fast propagating contractions shown in **A**. (**D**) Intraluminal pressure profile over time, from **C**.

Fast propagating contractions often occurred in clusters and the frequency ranged from 14.8 to 26.1 cycles/min (mean 19.7 ± 4.3 cycles/min), the cluster frequency was 1.7 ± 0.5 cycles/min (Fig. 4A) (N = 6). The decrease in diameter was 0.3 ± 0.1 cm. The propagation velocity was 2.9 ± 0.6 cm/s but varied over time and along the colon. Retrograde propagation often occurred in the distal region of the colon with a velocity of 4.6 ± 1.6 cm/s. Pressure activity occurred at the same frequency as the clusters, not at the frequency of the fast propagating contractions (Fig. 4C); however, no propagation velocity could be measured in the pressure maps. The intraluminal pressure value was 7.2 ± 1.7 cmH_2_O (Fig. 4B and D).

**Figure 5:**
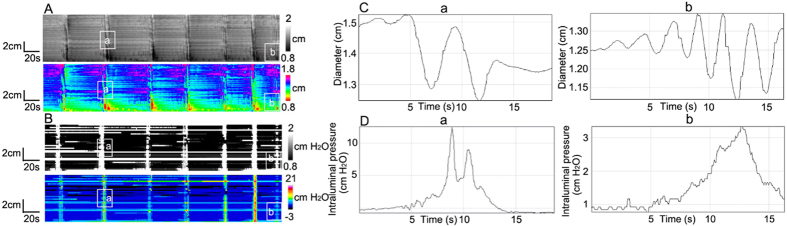
Clusters of fast propagating contractions. (**A**) Spatiotemporal diameter maps of clusters of fast propagating contractions. The boxed areas a and b show two clusters with different contraction frequencies and force. (**B**) Pressure maps associated with the fast propagating contractions in **A**. The boxed areas a and b were at the same point of colon as shown in a and b of **A**. (**C**) a,b: Amplitude over time profiles of boxes a and b in **A**. (**D**) a,b: Intraluminal pressure profiles of boxes a and b in **B**.

In most cases, the intraluminal pressure changes generated by a single fast propagating contraction could not be detected specifically, only a jagged pattern was seen throughout, but there were exceptions. As shown in the Fig. 5, two peaks in the diameter map (Fig. 5Ca) correlated with two peaks in the pressure map (Fig. 5Da). In another cluster, 5 peaks were observed in the diameter map (Fig. 5Cb), but the plot profile of the pressure map was unable to pick these up, the pressure activities merged (Fig. 5Db). When the force of contraction was strong enough, the pressure sensors caught rhythmic changes. With high frequencies and relatively low force, quick diameter changes did not result in pressure changes at this same frequency.

### Pressure patterns as a consequence of haustral boundary contractions

**Figure 6:**
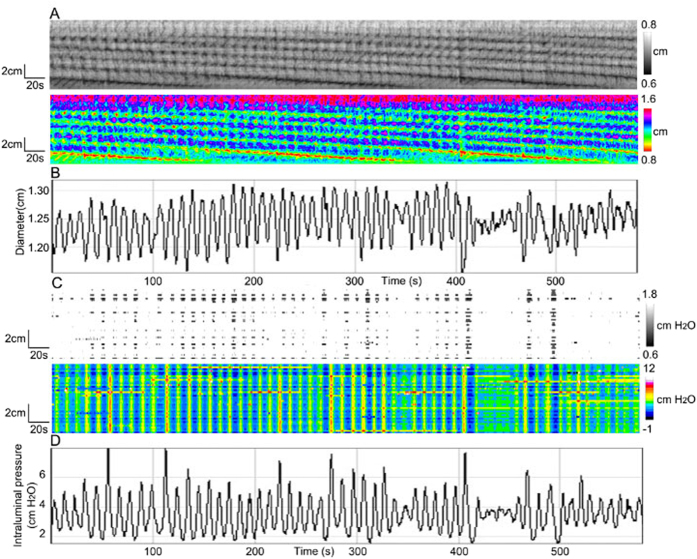
Haustral boundary contractions. (**A**) Spatiotemporal diameter maps of haustral boundary contractions interacting with ripples show slow propagation at a frequency of 0.5 ± 0.2 cycles/min in anal direction. (**B**) Amplitude over time profile of the whole rabbit proximal colon, from **A**. (**C**) Intraluminal pressure patterns as a consequence of haustral boundary contractions in **A**. (**D**) Intraluminal pressure profile of the whole proximal colon over time, from **C**.

Haustral boundary contractions are circular muscle ring contractions that propagate very slowly and divide the colon into haustra^9^,^10^. In the present study, the frequency of haustral boundary contractions ranged from 0.35 cycles/min to 0.75 cycles/min (mean 0.5 ± 0.2 cycles/min) and the antegrade propagation velocity was 7.8 ± 0.5 mm/s (N = 4). Haustral boundary contractions always interacted with ripples, and caused them to show an on/off/on/off pattern of contraction, resulting in rhythmic contractions at the ripple frequency (Fig. 6A). However, the color pressure map showed a pattern that was dominated by rhythmic simultaneous pressure waves at the ripple frequency, but the haustral boundary contractions were not evident (Fig. 6C). Interestingly, the gray scale pressure map of haustral boundary contractions showed the on off pattern caused by the collisions with the ripples. The black spots are relaxations relative to the white areas. When the ripples and haustral boundary contractions met, the summated amplitude increased; hence the black spots are the spots where the ripples are not increased in amplitude due to collision with haustral boundary contractions. If there were only ripples, as shown in the Fig. 3C, consecutive white and black vertical lines going across the pressure map would be seen. The diameter reduction when the two motor patterns coexisted was 0.21 ± 0.05 cm and the intraluminal pressure value was 2.0 ± 0.4 cmH_2_O.

**Figure 7:**
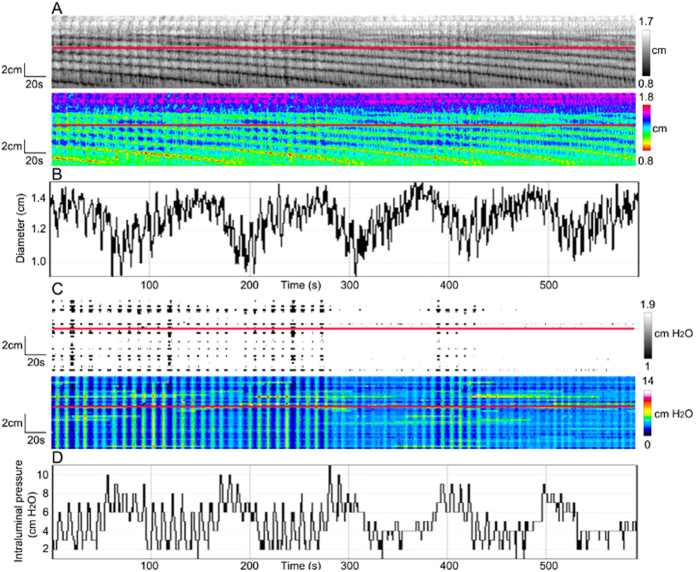
Haustral boundary contractions interacting with ripples. (**A**) Spatiotemporal maps of haustral boundary contractions occurred with ripples. (**B**) Amplitude profile along the red line in **A** showing an on/off/on/off pattern at the ripple frequency but also a low frequency oscillation at the haustral boundary contraction frequency. (**C**) Pressure patterns of haustral boundary contractions. The red line was set at the same point of colon as the red line in **A**. (**D**) Intraluminal pressure profile along the red line in **C** showing the ripple frequency as well as teh haustral boundary contraction frequency.

As shown in Fig. 7A and C, when a line was drawn through diameter maps and pressure maps at the same colon position, the plot profile (Fig. 7B) showed a rhythmic waxing and waning pattern at the haustral boundary contraction frequency. A similar pattern was present in the related pressure maps although propagation of the haustral boundary contractions could not be seen in the pressure maps.

### A change in motor pattern from fast propagating contractions to ripples

**Figure 8:**
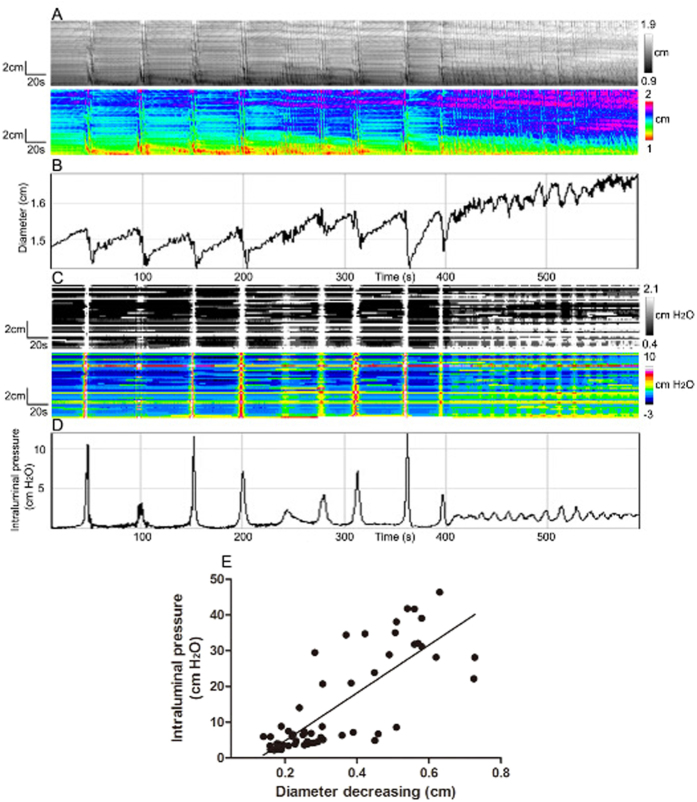
Correlations between motor patterns and intraluminal pressure when motor patterns change. (**A**) Spatiotemporal maps of clusters of fast propagating contractions replaced by ripples (**B**) Amplitude over time profile of the whole rabbit proximal colon, from **A**. (**C**) Pressure patterns as a consequence the contraction patterns in **A**. (**D**) Intraluminal pressure profile of the whole proximal colon over time, from **C**. (**E**) Correlation between intraluminal pressure and decrease in diameter (contraction).

The correlation between motor patterns and intraluminal pressure was further illustrated by following the change in motor pattern from fast propagating contractions to ripples (Fig. 8). The varying frequency and amplitude of the contractions were faithfully represented in the pressure profiles.

Twenty single waves of ripples and LDCs and fast propagating contractions from both spatiotemporal maps and pressure maps were selected randomly for correlation analysis. As shown in Fig. 8E, a positive correlation existed between decreases in diameter and the amplitude of the pressure transient (r = 0.800, p < 0.0001); the stronger the contraction the higher the intraluminal pressure.

## Discussion

The present study shows that within a certain range of contraction amplitudes and frequencies, the intraluminal pressure pattern faithfully resembles the contraction pattern. However, when the frequency is very high (as in fast propagating contractions in a cluster) the resultant intraluminal pressures merge. When the contraction speed of propagation is very fast (above ~5 cm/s), the resulting pressure occurs simultaneous. It should be noted that when the propagation velocity is high, the resolution of the manometry system may not be high enough to register the velocity and the patterns may appear simultaneous. The results of this study show that caution has to be exercised in the interpretation of manometry data with respect to assuming the motor patterns that underlie the pressure activities observed.

The results of the present study make it very likely that the simultaneous pressure waves observed in the *human* colon are also generated by very fast propagating contractions^3^,^11^,^12^. Occurrence of fast propagating contractions in the human colon is also supported by the occurrence of fast propagating electrical activities^13^ as well as visual observance of very fast transit using X-rays^14^. This means that the simultaneous pressure waves in the human colon should be regarded as one of the propulsive motor patterns. The current studies were done in a fluid filled colon; the motor patterns might be different compared to normal *in vivo* conditions, although *in vivo*, the most proximal part of the colon receives fluid from the intestine. In any case, the conditions of our experiments were similar to the human colon under conditions of high resolution manometry which is conducted in a cleaned colon, either also filled with water when a water-perfused catheter is used (as done currently by us) or completely empty when solid state or fiber-optics catheters are used.

The haustral boundary contractions that are such a characteristic motor pattern of the rabbit colon were not accurately reflected in the pressure maps. This is likely due to their low amplitude and very low propagation velocity. The assumption is that the slow contraction resulted in slow pressure changes that dissipated quickly in the relatively large colon cavity. Nevertheless, certain features of this motor pattern were observed. First, the haustral boundary contractions caused intermittent increase in ripple amplitude when the haustral boundary contraction summated with that of the ripple. This on/off pattern was clearly visualized. Second, the haustral boundary contractions showed a waxing and waning amplitude when followed over time in one particular spot on the colon, which was reflected in the pressure map.

The colon of the herbivorous rabbit is only a model for the omnivorous human colon and will not be identical. Future studies in the colon of the omnivorous pig are warranted to confirm the present results. Importantly, very fast propagating contractions, up to 16.7 cm/s, were observed in mini-pigs and they were associated with sphincter relaxation and gas expulsion^15^, similar to such occurrences in the human colon^3^,^11^,^16^,^17^.

What is the basis for very fast propagating contractions or the equally fast propagation of electrical activity that underlies the contractions? Since nerve action potentials travel fast, they might orchestrate fast contractions. However, contractions that are orchestrated by the extrinsic or intrinsic nervous systems do not follow the speed of nerve action potentials, they follow a programmed sequence of neural activity. Examples of neurally-driven propagating contractions are the vagal orchestration of esophageal peristalsis in human at 2–4 cm/s^18^,^19^ and the neurally driven migrating motor complex in the small intestine of humans that travels only at 5 cm/min^20^. It is likely that the fast propagating contractions are not dependent on nerves as was proven in the rabbit colon, where Lentle *et al*. suggested them to be associated with interstitial cells of Cajal associated with the myenteric plexus (ICC-MP)^9^. Slow wave driven propulsive contractions can travel at any speed and can switch direction instantaneously^21^. This is because the slow waves are transferred to the smooth muscle layers from a vast network of pacemaker cells, the interstitial cells of Cajal (ICC)^22^,^23^,^24^,^25^. The ICC network is in fact a network of coupled oscillators^21^,^26^,^27^,^28^,^29^,^30^. In the ICC-MP network, every ICC is active, always produces pacemaker activity and is electrically coupled to its neighbours. The slow waves are synchronized within the network, which has a gradient of intrinsic pacemaker frequencies going oral to anal. The result of the interaction (coupling) between oscillators is that waves can appear to propagate, although this only represents a coordinated phase difference between oscillators.

The speed of apparent propagation within this ICC network is determined by network properties, e.g. gap junction conductance and the slow wave frequency gradient, and can switch between antegrade, retrograde and simultaneous just based on subtle changes in the network properties^21^,^28^,^29^,^31^.

## Methods

All experiments were approved by the Animal Research Ethics Board at McMaster University (Animal Utilization Protocol 14-12-49). All methods were performed in accordance with the guidelines and regulations of the Animal Research Ethics Board of McMaster University and the Canadian Institutes of Health Research. In all experiments, the 3-taeniated proximal colon was removed from male or female adult New Zealand white rabbits (N = 18) of 1.8–2.5 kg body weight, anesthetized with 3% pentobarbital sodium injected into a marginal ear vein. After a midline incision, the whole proximal colon was removed and placed in warmed (37 °C) and continuously oxygenated (5%CO_2_ and 95%O_2_) Krebs solution (pH 7.3~7.4) which consisted of (mM) NaCl 118.1, KCl 4.8, NaHCO_3_ 25, NaH_2_PO_4_ 1.3, MgCl_2_ ∙ 6H_2_O 1.2, Glucose 12.2 and CaCl_2_ 2.5. The rabbit was euthanized thereafter by injecting air into the ear vein. The colon contents were gently washed out with warmed Krebs solution and external connective tissue was removed to provide high quality diameter maps. The whole proximal colon, about 14 cm long was mounted in an organ bath using an experimental setup that has been described previously^10^ and a Microsoft^®^ video camera was mounted above the preparation to record motility. Data acquisition occurred using Microsoft LifeCam software. Video recordings were analyzed with Image J aided by plugins written by Dr. Sean Parsons^10^. Spatiotemporal maps were created as D-maps quantifying *diameter* changes over time (created by circular muscle contraction). Two kinds of spatiotemporal maps were created with Image J, gray scale and 16 colour spectrum maps. Black represents relaxations in gray maps and red represents contractions in spectrum maps. Colon width was calculated at each point along the colon’s length (image Y-axis), for each video frame (image X-axis).

A custom designed silicone high-resolution water-perfused catheter was used, 41 sensors, 0.25 cm apart, hence the length of measurement was 10 cm (CR4-1109; Mui Scientific Mississauga Canada). The outer diameter was 4.0 mm. The catheter was placed in the colon through the proximal end and data acquisition was performed on a Medical Measurements System (MMS) platform (Enschede, the Netherlands). The colon was tied to the catheter at the most proximal end such that the catheter did not move during the experiments. The MMS system gave 200 mbar water pressure into the perfusion system resulting in very low flow rate. Changes in intraluminal pressure were prevented by keeping the level of water in the outflow cylinder constant. Two kinds of pressure maps were created with Image J, gray scale and 16-color pressure maps. Intraluminal pressure (coded as image intensity) was detected by each pressure sensor placed along the colon (image Y-axis). N = number of animals, n = number of patterns analyzed; values are expressed as mean ± SD.

## Additional Information

**How to cite this article**: Quan, X. *et al*. Relationships between motor patterns and intraluminal pressure in the 3-taeniated proximal colon of the rabbit. *Sci. Rep.* **7**, 42293; doi: 10.1038/srep42293 (2017).

**Publisher's note:** Springer Nature remains neutral with regard to jurisdictional claims in published maps and institutional affiliations.
